# Locations and characteristics of pharmacy deserts in the United States: a geospatial study

**DOI:** 10.1093/haschl/qxae035

**Published:** 2024-03-16

**Authors:** Rachel Wittenauer, Parth D Shah, Jennifer L Bacci, Andy Stergachis

**Affiliations:** CHOICE Institute, School of Pharmacy, University of Washington, Seattle, WA 98195, United States; Hutchinson Institute for Cancer Outcomes Research (HICOR), Fred Hutchinson Cancer Center, Seattle, WA 98109, United States; CHOICE Institute, School of Pharmacy, University of Washington, Seattle, WA 98195, United States; CHOICE Institute, School of Pharmacy, University of Washington, Seattle, WA 98195, United States; Department of Global Health, School of Public Health, University of Washington, Seattle, WA 98105, United States

**Keywords:** pharmacy desert, pharmacy access, pharmacy health services, pharmaco-equity

## Abstract

Pharmacies are important health care access points, but no national map currently exists of where pharmacy deserts are located. This cross-sectional study used pharmacy address data and Census Bureau surveys to define pharmacy deserts at the census tract level in all 50 US states and the District of Columbia. We also compared sociodemographic characteristics of pharmacy desert vs non-pharmacy desert communities. Nationally, 15.8 million (4.7%) of all people in the United States live in pharmacy deserts, spanning urban and rural settings in all 50 states. On average, communities that are pharmacy deserts have a higher proportion of people who have a high school education or less, have no health insurance, have low self-reported English ability, have an ambulatory disability, and identify as a racial or ethnic minority. While, on average, pharmacies are the most accessible health care setting in the United States, many people still do not have access to them. Further, the people living in pharmacy deserts are often marginalized groups who have historically faced structural barriers to health care. This study demonstrates a need to improve access to pharmacies and pharmacy services to advance health equity.

## Introduction

Community pharmacies are a crucial component of health care infrastructure in the United States.^1,2^ Nearly 7 in 10 adults between 40 and 79 years old take at least 1 prescription drug, and approximately 1 in 5 adults take 5 or more prescription drugs.^3^ In addition to dispensing medications for acute and chronic illnesses, a wide variety of health services are offered at pharmacies, including routine vaccination, opioid and addiction management therapy, contraception, and patient counseling on medications. There is a long history of pharmacists providing important community health services, and the scope and breadth of these practices have expanded over time.^4-6^ Recently, the COVID-19 pandemic highlighted the importance of community pharmacists and pharmacies as points of access for providing essential health products and services, including administering half of all COVID-19 vaccines.^7^

Communities that are both low-income and have low access to pharmacies are known as “pharmacy deserts” and lack access to the wide range of services provided at pharmacies.^8^ Pharmacy deserts disproportionately exist in rural or historically marginalized neighborhoods, compounding already-existing health inequities experienced by these communities.^8-16^ However, information is lacking on the specific locations and characteristics of pharmacy deserts nationwide, making it difficult to measure both the detrimental effects of poor access and the beneficial effects of pharmacy-based health services across geography.

The term *pharmacy desert* was first coined in 2014 in Qato et al's analysis of pharmacy desert locations and characteristics in Chicago.^8^ Since then, literature on this topic has grown to include identification of pharmacy deserts in Los Angeles County, the 30 largest metropolitan areas of the United States, and all rural counties throughout the United States.^8,10-13,16-20^ Additional analyses examine characteristics of pharmacy closures^9^ and several examine access to pharmacies but from an exclusively spatial lens (ie, not considering income factors).^2,21^ While these piecemeal analyses are beginning to create a cohesive evidence base, each map uses different methods and data sources and applies them to a small geographic area. To date, there is no comprehensive, systematically defined map of pharmacy desert locations in the United States. The objective of this study was to identify the locations of pharmacy desert neighborhoods using a standard methodology and characterize the population that lives in these areas and the pharmacies that serve them.

## Data and methods

### Approach

We applied the pharmacy desert definition^8^ to classify the census tracts in all 50 US states and the District of Columbia. We reported locations and population estimates of all pharmacy deserts and presented them visually on a choropleth map. We examined the tract-level social and demographic characteristics of populations (eg, race/ethnicity, health insurance status) in pharmacy desert communities vs non-pharmacy desert communities and the characteristics of pharmacies (eg, services offered, independent vs chain ownership) serving pharmacy desert communities vs those serving non-pharmacy desert communities.

### Data sources

The locations and characteristics of all licensed pharmacies as of April 2022 were sourced from the National Council for Prescription Drug Programs.^22^ Data to define pharmacy deserts and their associated characteristics were from multiple sources produced by the US Census Bureau, including the 2020 Decennial Census and the 2017–2021 5-year American Community Survey.^23,24^ Additional detail on the dataset creation process, variable definitions, and the calculation process can be found in Supplement A1. (To access the Appendix, click on the Details tab of the article online.)

### Key measures

We created binary indicators for the low-income and the low-access criteria of the pharmacy desert definition. A census tract meeting both indicators was then designated as a pharmacy desert. The specific measures are defined as follows:

1. Low income: Tract has either (1) 20% or more of its population living below the Federal Poverty Level or (2) a median household income that was less than 80% of the median income of the nearest metropolitan area.
2. Low access: Tract has at least 33% of its population living 1 mile or more from the pharmacy for urban tracts, more than 5 miles for suburban tracts, more than 10 miles for rural tracts, and more than 0.5 miles for tracts with less than 100 individuals owning a car.

Census tracts were classified as urban, suburban, and rural using population density,^21^ based on thresholds defined in previous analyses^25^ and consistent with survey research on living environments.^26^

We used areal interpolation at the block level to calculate the proportion of a tract's population living within the accessibility radius of a pharmacy.^27^ For each urbanicity level's acceptable pharmacy radius (0.5, 1, 5, or 10 miles), we evaluated whether the centroid of every census block in the United States was inside or outside of a pharmacy's radius. Then, for every tract, we summed the population of the blocks that were inside any pharmacy radius based on tract urbanicity. If more than one-third of the population was not in the radius, the tract was designated as meeting the “low access” component of the pharmacy desert definition. Additional details on this process are available in Supplement A1.

### Statistical analyses

We compared characteristics of populations and pharmacies in pharmacy desert vs non–pharmacy desert communities. For continuous variables we tested statistical significance with a 2-sample *t* test and for categorical variables a chi-square test of independence. All *P* values were corrected for multiple comparisons using the Benjamini-Hochberg correction.^28-30^ All analyses were conducted using R version 4.2.2 (R Foundation for Statistical Computing, Vienna, Austria). Maps were created using Tableau Desktop version 2023.2 (Tableau Software LLC, Seattle, USA) (see Supplement A1 for additional details).^31,32^

## Results

### Locating and quantifying pharmacy deserts

Nationally, 15.82 million (4.7%) of all people in the United States live in pharmacy desert communities. Of the 84 414 census tracts, 4679 (5.5%) were identified to be pharmacy deserts, 78 723 (93.3%) were designated as not pharmacy deserts, and 1012 could not be classified due to no available income data (*n* = 446, 0.5%) or having a population of zero (*n* = 566, 0.7%). Among the census tracts that are pharmacy deserts, the majority have zero pharmacies (*n* = 4,421, 94.5%) (Table 1). Of the 60 475 community pharmacies nationwide, only 294 (0.5%) are serving pharmacy desert communities (Table 2).

**Table 1.** Characteristics of populations living in pharmacy deserts vs non-pharmacy deserts.

	Pharmacy desert (*n* = 4679)	Not a pharmacy desert (*n* = 78 723)
Urbanicity and access, *n* (%)		
Urbanicity of census tract^a^		
Urban	2692 (57.5%)	19 972 (25.4%)
Suburban	204 (4.4%)	30 264 (38.4%)
Rural	1783 (38.1%)	28 487 (36.2%)
Pharmacies per census tract^a^		
Zero pharmacies in the tract	4421 (94.5%)	43 249 (54.9%)
One pharmacy in the tract	34 (0.7%)	14 852 (18.9%)
Two or more pharmacies	224 (4.8%)	20 622 (26.2%)
Social characteristics, mean (SD)		
Prop. below FPL^a^	24.6% (14.3)	13.0% (10.8)
Median household income^a^	$46 400 ($14 900)	$76 000 ($37 000)
Prop. with high school education or less^a^	33.2% (10.4)	27.6% (11.4)
Prop. with no health insurance^a^	15.2% (11.2)	9.89% (8.56)
Prop. with public health insurance^a^	41.9% (15.8)	35.4% (12.9)
Prop. that do not speak English^a^	5.73% (9.53)	3.07% (6.78)
Demographic characteristics, mean (SD)		
Prop. with ambulatory disability^a^	10.4% (6.37)	8.34% (4.97)
Prop. older adults (age 65+)^a^	15.0% (10.1)	16.9% (8.80)
Race and ethnicity		
Prop. NH, White^a^	45.5% (32.1)	61.2% (29.3)
Prop. NH, Black^a^	19.1% (26.1)	12.6% (20.4)
Prop. NH, Asian^a^	3.29% (7.03)	5.25% (9.80)
Prop. NH, AIAN^a^	3.50% (1.49)	0.51% (2.78)
Prop. NH, 2 or more races^a^	3.02% (3.31)	3.16 (3.16)
Prop. NH, other race	0.33% (1.02)	0.36% (1.10)
Prop. Hispanic, White race^a^	12.0% (16.1)	7.97% (11.6)
Prop. Hispanic, Black race^a^	0.39% (1.11)	0.35% (1.15)
Prop. Hispanic, Asian race	0.06% (0.29)	0.07% (0.37)
Prop. Hispanic, AIAN race^a^	0.42% (1.28)	0.22% (0.80)
Prop. Hispanic, 2 or more races^a^	4.74% (6.85)	3.47% (5.37)
Prop. Hispanic, other race^a^	7.32% (11.5)	4.70% (8.80)

Abbreviations: AIAN, American Indian or Alaskan Native; FPL, Federal Poverty Level; NH, non-Hispanic; Prop., proportion of tract population; SD, standard deviation.

Source: Authors’ analysis of pharmacy address data, 2020 Decennial Census data, and 2021 5-year American Community Survey data.

^a^Denotes statistically significant difference of characteristic in pharmacy desert vs non-pharmacy desert tracts at *P<* .01. All *P* values are from a *t* test for continuous variables or a chi-square test for categorical variables, all adjusted for multiple comparisons using the Benjamini-Hochberg correction.

**Table 2.** Characteristics of pharmacies located in pharmacy deserts.

	Pharmacy desert (*n* = 294)	Not a pharmacy desert (*n* = 60 175)	*P* ^a^
Urbanicity of pharmacy location			
Urban	179 (60.9%)	15 986 (26.6%)	<.001
Suburban	8 (2.7%)	26 922 (44.7%)	
Rural	107 (36.4%)	17 206 (28.6%)	
NA: no population	0 (0%)	61 (0.1%)	
Pharmacy ownership			
Independent	121 (41.2%)	22 010 (36.6%)	<.001
Chain	165 (56.1%)	37 371 (62.1%)	
Franchise	3 (1.0%)	659 (1.1%)	
Government	5 (1.7%)	135 (0.2%)	
Immunization services available	221 (75.2%)	48 510 (80.6%)	.051
ADA accessible	290 (98.6%)	59 429 (98.8%)	1.000
Multidose packaging available	73 (24.8%)	12 056 (20.0%)	.087
Emergency services 24 hours	82 (27.9%)	17 841 (29.6%)	.621
Walk-in clinic available	40 (13.6%)	3923 (6.5%)	<.001
Compounding pharmacy	172 (58.5%)	37 230 (61.9%)	.335
DME available	219 (74.5%)	46 742 (77.7%)	.323

Abbreviations: ADA, Americans with Disabilities Act; DME, durable medical equipment; NA, not applicable.

Source: Authors’ analysis of pharmacy address data, 2020 Decennial Census data, and 2021 5-year American Community Survey data.

^a^All *P* values result from a *t* test for continuous variables and a chi-square test for categorical variables, all adjusted for multiple comparisons using the Benjamini-Hochberg correction.

The states with the highest proportion of their adult population living in pharmacy deserts are New Mexico (14.9%), Alaska (14.8%), and Arizona (8.6%). The states with the lowest proportion were New Hampshire (0.86%), New Jersey (1.05%), and New York (1.74%). The states with the highest number of adults living in pharmacy deserts are California (2.53 million), Texas (1.68 million), and Florida (749 103), reflecting the size of those states’ large populations. These data by state are summarized in Supplement Figure A2-1.

**Figure 1.**
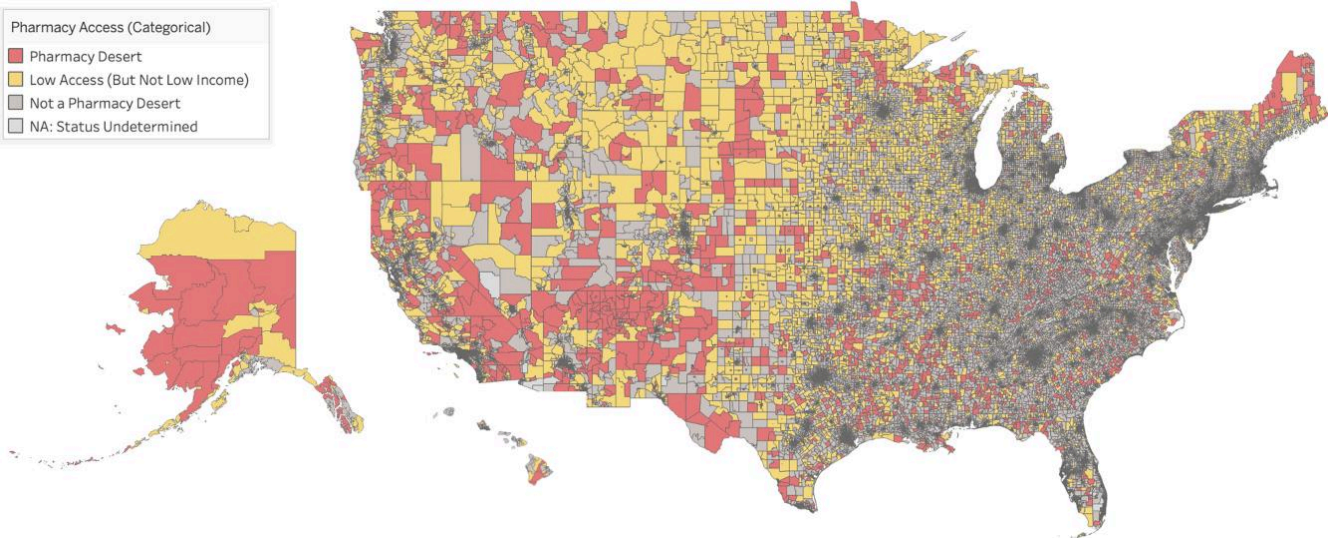
Choropleth map of pharmacy deserts and pharmacy access at the census tract level in the United States in 2022. Maps of select urban areas are available in Supplemental Figure A2-3. For more detail, an interactive Tableau version of this map is available at https://tinyurl.com/PharmacyDesertsMap2022. Source: Authors’ analysis of pharmacy address data, 2020 Decennial Census data, and 2021 5-year American Community Survey data.

The locations of all pharmacy desert tracts and all tracts with low access (but which did not meet the low-income criteria and thus are not considered full “pharmacy deserts” in our main analysis) are visualized on a choropleth map in Figure 1. An interactive version of the map is available via Tableau Public (https://tinyurl.com/PharmacyDesertsMap2022). Map inset views of 5 example urban areas in the United States are available in Supplement Figure A2-3. Locations of each individual pharmacy and the number of pharmacies per population are also mapped in Supplement Figures A2-4 and A2-5.

If the low-income component of the pharmacy desert definition was not accounted for, as is the case in some pharmacy access research,^2,21,33,34^ then a higher number of census tracts would be designated as pharmacy deserts. In this case, beyond the 4679 pharmacy desert tracts, an additional 7536 tracts were identified as having low access to pharmacies. This total of 12 215 tracts represents approximately 34 million adults (13.2% of all adults) in the country living in areas with low access to pharmacies.

### Characteristics of pharmacy deserts

Pharmacy deserts are most often located in urban and rural areas as opposed to suburban areas (*P* < .001). Communities that are pharmacy deserts, as compared with non-pharmacy desert communities, have a higher proportion of people who have a high school education or less (33.2% vs 27.6%, *P* < .001), have no health insurance (15.2% vs 9.9%, *P* < .001), have public health insurance (41.9% vs 35.4%, *P* < .001), speak English “not well” or “not at all” (5.7% vs 3.1%, *P* < .001), have an ambulatory disability (10.4% vs 8.3%, *P* < .001), identify as non-Hispanic Black race (19.1% vs 12.6%, *P* < .001), identify as American Indian or Alaskan Native (3.5% vs 0.5%, *P* < .001), and identify as Hispanic White race (12.0% vs 8.0%, *P* < .001). Pharmacy desert communities have a lower proportion of people who are under the age of 65 (15% vs 16.9%, *P* < .001), identify as non-Hispanic White race (45.5% vs 61.2%, *P* < .001), and identify as non-Hispanic Asian race (3.3% vs 5.3%, *P* < .001). These results are summarized in Table 1. Most patterns seen at the national level persist when stratified by urbanicity—for example, pharmacy deserts still have a higher proportion of people with lower education, non-White race, and no health insurance across urban, suburban, and rural areas. However, among rural pharmacy deserts vs rural non–pharmacy deserts, there is a slightly higher proportion of individuals aged 65 years and older. Supplement Table A2-2 presents these data stratified by urbanicity level and Supplement Table A2-3 presents these data for tracts that have low spatial access to pharmacies but did not meet the low-income criteria of the pharmacy desert definition.

We also examined the characteristics of pharmacies that are located in pharmacy desert tracts vs non-pharmacy desert tracts (Table 2). Pharmacies serving pharmacy deserts are more often in urban and rural areas rather than suburban areas. Pharmacies located in pharmacy deserts vs non-pharmacy desert areas have different distributions of ownership: those in pharmacy deserts are more often independently owned pharmacies (41.2% vs 36.6%, *P* < .001). Pharmacies in pharmacy deserts also have varying levels of service offerings: for example, they more often have walk-in clinics available as compared with non-pharmacy desert pharmacies (13.6% vs 6.5%, *P* < .001). Additional detail is available in Supplement Table A2-4.

## Discussion

Our study provides estimates of the number of adults living in pharmacy deserts in the United States—nearly 16 million people. The proportion and number of adults living in pharmacy deserts vary by state, from less than 1% of adults in New Hampshire to nearly 15% of adults in New Mexico. Nationally, our results indicate that pharmacy desert neighborhoods are associated with many social and political determinants of health, including lower educational attainment, lower income, lower health insurance coverage, and a higher proportion of people identifying as racial or ethnic minorities. These patterns did not vary substantially by urbanicity of the neighborhood. Of the tracts that are pharmacy deserts, nearly 95% had contained zero pharmacies. In examining characteristics of pharmacies, we found that the few that are serving pharmacy desert communities are more often independently owned rather than chain owned, and more often have walk-in clinic services available as compared with pharmacies serving non-pharmacy deserts.

Compared with other studies of pharmacy deserts with similar operational definitions, our results indicate a smaller proportion of the population is living in pharmacy deserts. This is potentially due to our paper's inclusion of a “suburban” category rather than an urban/rural binary, which we believe to be an important distinction but is not often included. An analysis of pharmacy deserts in Washington State with a similar approach (but using only urban and rural categories) found 8% of the adult population living in pharmacy deserts vs our analysis that found 7.62% of Washington adults were in pharmacy deserts.^19^ An analysis of pharmacy deserts in the 30 largest metropolitan areas in the United States found that 32% of tracts were pharmacy deserts, while our study found that 11.8% of urban tracts were pharmacy deserts.^18^ We believe that using a 5-mile radius for tracts with less than 5000 people per square mile (suburban definition) is a key contributor to this discrepancy, as is our study's limitation of using linear distance rather than road distance. The magnitude of the population living in pharmacy deserts in our study is comparable to that of people living in food deserts (similar in both concept and definition): approximately 18.8 million people (6.1% of the US population) live in census tracts that are food deserts.^35^

In terms of population characteristics, other published studies of pharmacy deserts in smaller geographies have found similar associations.^20^ For example, studies of pharmacy access in Shelby County, Tennessee, Chicago Illinois, and the 30 most populous US cities found that neighborhoods with worse pharmacy access were associated with higher proportions of racial and ethnic minority groups.^8,12,18^ In Los Angeles County, a clustering analysis identified 2 statistically distinct types of pharmacy deserts: 1 type with higher population density, a younger population, and a higher proportion of racial/ethnic minorities, and a second type with a lower population density and a higher proportion of non-Hispanic White persons.^10^ The results of this national analysis align with those findings from Los Angeles County.

### Implications

These findings have implications for patient and population health, policy solutions, and future research to understand and address patient access to medications and pharmacy services. We note that, while many insurance plans provide their enrollees with access to prescriptions via mail order, the focus of this analysis is access to physical pharmacy locations that provide many important health services including and beyond dispensing medications. Implications of this study's findings regarding lack of access to pharmacy-based services are summarized in Table 3 and further described below.

**Table 3.** Implications of study results for patient and population health, policy solutions, and future research.

	Key findings	Implications
Application to patient and population health	Nearly 16 million adults in the United States live in pharmacy deserts People living in pharmacy deserts already face many known barriers to accessing health care including lower educational level, lower self-reported English-speaking ability, and lower insurance coverage Pharmacy desert communities have a higher population of racial and ethnic minorities as compared with non-pharmacy deserts	Many adults in the United States do not have adequate access to pharmacies, which (1) signals gaps in access to important routine health care, (2) weakens emergency and pandemic preparedness infrastructure, and (3) raises health equity concerns. Left unaddressed, pharmacy deserts may contribute to widening health disparities for individuals living in these areas
Policies to improve access to pharmacies	Nearly 95% of pharmacy deserts have no pharmacies in them at all Pharmacy desert communities have a higher proportion of their population with no insurance and with public insurance compared with non-pharmacy desert communities Pharmacy deserts were overwhelmingly in lower-income areas and in urban or rural areas (rather than wealthier or suburban areas); evidence shows pharmacies in these areas are more likely to close Populations living in pharmacy deserts were more likely to have public health insurance and be under the age of 65—2 characteristics that point to the inability of state-level laws to adequately act through commercial insurance plans to improve pharmacy access for patients and reimbursements for pharmacy services	State and federal policies can halt the growth of pharmacy deserts and address patient care in existing ones by (1) preventing closures of existing pharmacies especially in underserved areas, possibly including PBM regulation and provider status recognition by CMS (2 issues that impact pharmacy revenue and viability), (2) establishing or incentivizing new services access points for patients similar to the HPSA incentives for primary care, and (3) leveraging new technology and care models such as telepharmacy, mobile clinics, and expanded scopes of practice New medicines, technologies, or policies that are intended to be delivered or act through pharmacies to improve patient health will have minimal effect in pharmacy desert neighborhoods; thus, it is essential to prioritize these areas in any policies which aim to improve access to health care via pharmacy-based services
Future research on pharmacy access	Inclusion of an income component in the pharmacy definition substantially reduces the population who are considered to be living in “pharmacy deserts,” as opposed to if only geographic access was considered Study has many limitations that could be improved upon with more robust methods and data in future research	The field of pharmacy-based services research would benefit from a carefully considered consensus definition on what a “pharmacy desert” is and how to parameterize their locations for analysis, particularly including the inclusion of an income component and role of urbanicity and access radius definitions The causes of pharmacy deserts and individual-level health effects on patients should be explored in future research

Abbreviations: CMS, Centers for Medicare and Medicaid Services; HPSA, Health Professional Shortage Area; PBM, pharmacy benefit manager.

Source: Authors’ analysis of pharmacy address data, 2020 Decennial Census data, and 2021 5-year American Community Survey data.

#### Implications for patient and population health

At a broad level, these results highlight that many adults in the United States do not have adequate access to pharmacies. This lack of access (1) signals important barriers in routine health care, (2) weakens emergency and pandemic preparedness infrastructure, and (3) raises health equity concerns.

Access to pharmacies and the variety of services pharmacists provide is an important means to protect and promote one's health. One cohort study found that, when neighborhood pharmacies closed, patients who had previously been filling their cardiovascular medication prescriptions in those stores had a clinically and statistically significant decline in medication adherence.^36^ Our results found that the most common structure of a pharmacy desert is to have no pharmacies in that neighborhood as opposed to inadequate access driven by per capita pharmacy to population ratio—a dynamic that poses a clear barrier for patients to access their medications and pharmacy services. Our results further found that, in communities that do have pharmacies, a smaller proportion of pharmacies in pharmacy deserts (compared with non-deserts) offered health services such as administering immunizations, selling durable medical equipment, and offering 24-hour emergency services. Although these differences were not statistically significant in our study, past analyses have identified statistically different service offerings in areas that are pharmacy deserts compared with non-deserts.^13,33^ Lack of access to pharmacies puts pharmacy desert communities—and their nearly 16 million residents—at a disadvantage for staying healthy.

Nationwide gaps in pharmacy accessibility are also noteworthy for emergency preparedness and response. The COVID-19 pandemic is the most recent example of the importance of pharmacy infrastructure—not only for testing, vaccinations, and treatment access to mitigate health and economic consequences of the pandemic but to enable continuity of care for peoples’ prescription medications.^7^ In climate-related and natural disasters, pharmacists remain trusted health providers who are perceived as responsible for the continuity of medication supply chains by both patients and other health care providers,^37,38^ and may be well suited to take on roles such as triaging evacuees, assessing vaccination needs, and providing over-the-counter and prescription medications.^39^ People living in pharmacy desert communities may be left behind in accessing important emergency services.

If left unaddressed, pharmacy deserts may contribute to widening health disparities for populations living in these areas. Our results indicate that, on average, pharmacy desert residents already face many known barriers to accessing health care, including lower income, lower educational level, lower English-speaking ability, and lower health insurance coverage. All of these barriers may layer on each other to further limit patients’ abilities to reach accessible, appropriate, affordable health care.^40^ Pharmacy desert communities also have a higher population of racial and ethnic minorities as compared with non-pharmacy deserts. For example, the proportion of population identifying as American Indian or Alaskan Native is 7 times higher in pharmacy deserts compared with non-pharmacy deserts (3.5% vs 0.5%), and the proportion of Black population is higher as well (19.1% vs 12.6%). The association between pharmacy locations today and race-based exclusionary policies from decades ago, such as redlining, has been established empirically,^41^ and the propagation of health disparities for historically minoritized groups will continue to propagate through the legacies of structural racism and colonialism if deliberate steps are not taken to close access gaps in these specific communities.^42,43^

#### Implications for policy

State and federal policies can halt the growth of pharmacy deserts and address patient care in existing ones by (1) preventing closures of existing pharmacies especially in underserved areas,^9^ (2) establishing or incentivizing new service access points for patients,^44,45^ and (3) leveraging new technology and care models to reach all patients (including adoption of tele-pharmacy, addressing staff shortages, using mobile clinics, and expanding scopes of practice).^33,46-49^ One important factor in pharmacy closures is the lack of a sufficient level of revenue. Pharmacies serving primarily low-income, publicly insured, and uninsured populations are at increased risk of closure, and independent pharmacies are more likely to close than chain pharmacies.^9^ Legislative efforts to acknowledge pharmacists as health care providers in Medicare,^44,50,51^ regulate pharmacy benefit managers,^52,53^ and provide Health Professional Shortage Area (HPSA)–like financial incentives for pharmacies operating in underserved areas could all help alleviate this financial pressure, particularly on pharmacies serving medically underresourced communities. The most effective policy solutions may take different forms for pharmacies in urban areas vs rural areas and regionally, as the logistics of reaching people in these areas differ greatly. Federal-level legislation is likely to have a more meaningful impact than state-level changes: the results of our study found that populations in pharmacy deserts were more likely to have public health insurance and be under the age of 65—2 characteristics that point to the inability of state-level laws to adequately act through only commercial insurance plan regulations to improve pharmacy access for patients.

We also found that the most common type of pharmacy desert were tracts with no pharmacies in them at all (94.5% of pharmacy deserts). New medicines, technologies, or policies that are intended to be delivered through pharmacies to improve patient health will undoubtedly have minimal availability and effect in pharmacy desert neighborhoods. Thus, it is essential to consider the differential impact in these communities of any policies that aim to improve access to health care via pharmacy-based services.^54^

#### Implications for future research

Beyond addressing this study's conceptual and methodological limitations, there are several ways that future research can extend this work. To inform ongoing policy discussions such as provider status legislation, the relationship between HPSAs and pharmacy deserts should be better defined. One study found that, within urban areas in the United States, the prevalence of pharmacy deserts was similar in Medically Underserved Areas (MUAs) vs non-MUAs, indicating that national measures of primary care access should not be presumed to correlate directly with pharmacy access.^18^ If a better understanding of these differences is to be accomplished, consensus in the field about an appropriate definition for low pharmacy access must first be achieved, including the inclusion of any income components and delineating access radii that are based on empirical evidence relating geographic accessibility to real-world patterns of pharmacy use and acceptability to patients.

Once pharmacy deserts are defined, evaluations of interventions and of patient health implications can take place. For example, an evaluation of any negative effects at the individual patient level of accessing medications in pharmacy deserts vs non-pharmacy deserts could help policymakers understand the magnitude and implications of these access concerns, and evaluations of the effects of varying state-level pharmacy policies (eg, regulation of pharmacy benefit managers, provider status, telemedicine, etc) can be contextualized. More robust statistical and causal inference methodologies can be applied to determine root causes of pharmacy deserts and may reveal potential solutions to prevent or reduce the loss of community pharmacies, and the vital services they provide, in the future.

### Limitations

The data underlying this analysis have several limitations. These pharmacy locations are as of April 2022 and may soon be outdated. In 2022 and 2023, the 3 largest pharmacy chains (CVS, Walgreens, and Rite Aid) all announced significant planned store closures numbering over 1500 total stores.^55^ Previous research has indicated that pharmacy closures are more likely to happen in low-income areas in both urban and rural settings,^9^ which the results from this analysis suggest already face higher likelihoods of being a pharmacy desert. These trends suggest a need to update this map in the coming years. The results presented here should thus be considered an underestimate of the gaps in pharmacy access.

These results are also limited by several assumptions in parameterizing pharmacy deserts, particularly in defining urbanicity and selecting access thresholds. Our use of population density as a proxy for urban status presents several challenges, we apply linear distance rather than travel time, and empirical data justifying the specific mileage of the access radii are limited. However, we note that minimum pharmacy benefit requirements from CMS also use population density to define urban, suburban, and rural areas and corresponding acceptable radii.^21,56^ Further limitations to this study can be found in Supplement A1.

Finally, there are contextual limitations to consider when interpreting these results. This analysis assumes that any patient can go to any pharmacy, but, in reality, insurance networks determine a select list of pharmacies for their enrollees. For any given individual, a map of pharmacies that accept their specific insurance would be much sparser. For example, in Washington State, pharmacies in rural areas were less likely to contract with Medicaid, heightening the existing access disparities there.^17^ In addition to insurance coverage, a long list of nonspatial factors feed into the concept of “access,” including language spoken, financial accessibility, hours and appointments, ADA (Americans with Disabilities Act) accessibility, quality of care, and more.^57^ These factors are well summarized by Levesque et al^40^ in their description of the interface of health systems and populations. The results of our study indicate that populations in pharmacy deserts have a higher proportion of individuals who speak English “not well” or “not at all,” have lower median incomes, and have more of their community living below the Federal Poverty Level. While describing access barriers comprehensively is outside the scope of this analysis, a spectrum of challenges must be considered by policymakers seeking to improve patient access to pharmacies.

### Strengths of the study

This analysis also has several strengths. To our knowledge, this study represents the first published estimates of locations and characteristics of pharmacy desert locations at the local level throughout the United States applying the most widely used definition of “pharmacy desert.” We use the precise location of all licensed community pharmacies and population count data from the 2020 Decennial Census—the most comprehensive population estimate available. We also derive proportions of the population living inside vs outside the radius of a pharmacy using areal interpolation with the most granular geographic unit possible—all 8.1 million census blocks—and incorporate suburban locations as a category beyond an urban/rural binary. Last, we include an interactive map of results and all source codes alongside our publication to facilitate further inquiry on the definition and characteristics of pharmacy deserts. Despite the limitations described above, these results summarize a comprehensive national estimate of pharmacy desert locations and characteristics.

## Conclusion

In this first national estimate of pharmacy desert locations and characteristics, 15.8 million people in the United States are living in areas without adequate access to pharmacies. These communities span all 50 states and the District of Columbia and occur in urban, suburban, and rural areas. Populations residing in pharmacy deserts are more likely to face multiple known social, political, and demographic barriers to accessing health, including lower educational level, lower health insurance coverage, a higher proportion identifying as racial or ethnic minorities, a higher proportion with difficulty speaking English, and a higher proportion with ambulatory disabilities. These findings raise important concerns about pharmacy services and medication access in these specific communities throughout the United States. Future research and policy solutions should consider pharmacy desert locations in evaluating pharmacy-based health services and improvements to medication access.

## Supplementary Material

qxae035_Supplementary_Data
