# Supplementary material for: Locations and characteristics of pharmacy deserts in the United States: a geospatial study
Source: Health Aff Sch. 2024 Mar 16;2(4):qxae035. doi: 10.1093/haschl/qxae035 (PMC11034534; doi:10.1093/haschl/qxae035)
Supplement: qxae035_Supplementary_Data [file qxae035_supplementary_data.zip › PharmacyDesertsManuscript2_SupplementalMaterial_vclean.docx]

Supplemental Appendix

Accompanying the study: “Locations and characteristics of pharmacy deserts in the United States: A geospatial study”

Table of Contents

[Supplement A1: Technical supplement 2](#_Toc160520678)

[1.1 Data sources 2](#_Toc160520679)

[1.2 Geographic calculations 4](#_Toc160520680)

[1.3 Accessing source data and analysis code 6](#_Toc160520681)

[1.4 Additional study limitations 6](#_Toc160520682)

[Supplement A2: Results supplement 8](#_Toc160520683)

[2.1 Population living in pharmacy deserts by state 8](#_Toc160520684)

[2.2 Pharmacy accessibility results 9](#_Toc160520685)

[2.3 Characteristics of populations and pharmacies in pharmacy deserts 12](#_Toc160520686)

[Supplemental Materials References 18](#_Toc160520687)

# Supplement A1: Technical supplement

## 1.1 Data sources

The locations and characteristics of all licensed pharmacies as of April 2022 were sourced from the National Council for Prescription Drug Programs (NCPDP). These data contained the physical address of each pharmacy as well as characteristics of the pharmacy and services offered, such as vaccination services. We specifically used the Provider Information and the Services Information tables available in NCPDP’s *dataQ™* v3.1 product, which aggregates data self-reported by each included pharmacy. The remaining data to define pharmacy deserts and their associated characteristics were from multiple sources produced by the Census Bureau, including the 2020 Decennial Census and the 2017-2021 5-year American Community Survey. All variable definitions are listed in Supplemental Table A1-1.

To create the census-tract level dataset, we used the Census Bureau API and the tidycensus R package to extract a data table containing population counts, land area, geographic boundaries, and census tract GEOID for all 84,414 census tracts in the 50 U.S. states and the District of Columbia sourced from the 2020 Decennial Census. We merged this table with a second table of census tract-level estimates derived from the 2021 5-year American Community Survey, which contained estimates of population social and demographic characteristics including race/ethnicity, education level, health insurance status, language spoken, and more as listed in Supplemental Table A1-1. The tract-level datasets were merged using the 11-digit tract GEOID.

To create the pharmacy dataset for analysis, the NCPDP pharmacy dataset (n=82,520 licensed pharmacies) was restricted to contain community pharmacies open to the public in all 50 states and the District of Columbia (e.g., excluding military pharmacies, alternate dispensing sites, etc.) (n=60,501 licensed pharmacies). We then geocoded (i.e., assigned latitude and longitude to) each pharmacy based on its physical address using the Google Maps API. Of the 60,501 pharmacies, 34 were not able to be read by Google Maps and were geocoded manually. An additional 26 pharmacies were not able to be geocoded successfully and were excluded from analysis. We used the st_intersects function of the “sf” package in R to evaluate each pharmacy point location’s intersection with a census tract polygon and thus assign each pharmacy to the census tract that it is located in. The full dataset creation process is summarized in Figure A1-1.

**Variable creation**

The low-income component of a pharmacy desert definition is partially based on if a tract has a median household income that was less than 80% of the median income of the nearest metropolitan area. If a tract was located in a census-defined Metropolitan Statistical Area (MSA), the median income of that MSA was used as the threshold. If the tract was not located in an MSA, the median income of the state was used as the threshold.

To describe characteristics of census tracts, we calculated the proportion of population with each characteristic by dividing the census estimate by the denominator of total population that was asked that question. For example, the proportion of adults with no health insurance was calculated by summing the number of adults with no health insurance by the denominator of adult population asked that census question. Binary and categorical versions were created of all pharmacy service characteristics from the NCPDP data using definitions provided by NCPDP.

Figure A1-1. Data sources and dataset creation


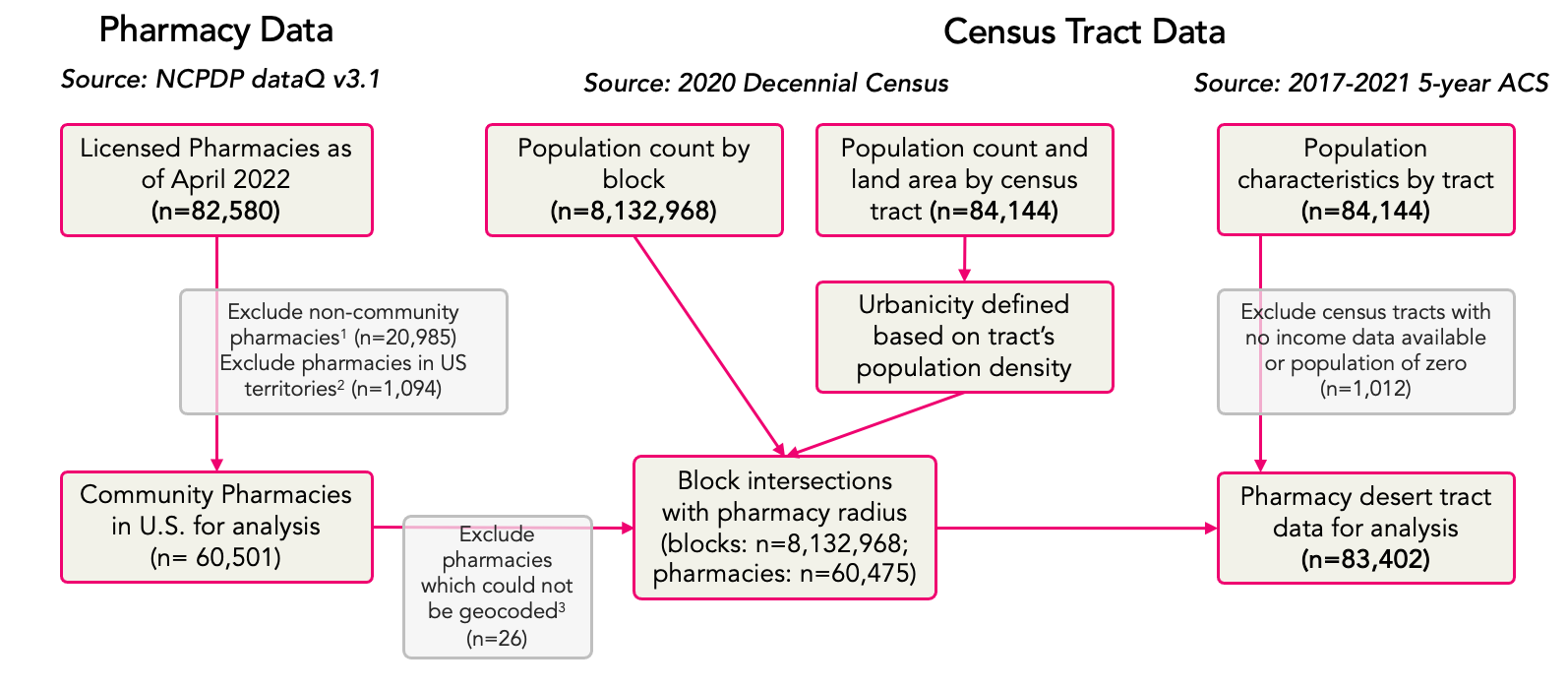


^1^ Pharmacy types that were excluded were: Non-pharmacy dispensing sites (n=8,833), Long-term care pharmacies (n=4,202), Closed Door Facilities (n=1,395), Institutional pharmacies (n=1,246), Clinic pharmacies (n=1,117), Home infusion therapy providers (n=771), Military pharmacies (n=697), Compounding pharmacies (n=662), Specialty pharmacies (n=628), Mail-order pharmacies (n=354), Indian Health Service pharmacies (n=305), Veterans Administration pharmacies (n=267), Alternate Dispensing Sites (n=163), Durable medical equipment pharmacy (n=161), Managed care organization pharmacies (n=141), Nuclear pharmacies (n=18), Parenteral nutrition (n=15), Nursing facility supplies (n=6), Oxygen equipment (n=2), Customized equipment (n=1), Dialysis equipment (n=1).

^2^ Pharmacies excluded from US territories were: Puerto Rico (n=1041), Guam (n=24), Virgin Islands (n=20), Northern Mariana Islands (n=9)

^3^ Note: 34 observations were unable to be geocoded by Google Maps API and were added manually, an additional 26 could not be geocoded

Abbreviations: ACS: American Community Survey; API: Application Programming Interface; D.C.: District of Columbia; NCPDP: National Council for Prescription Drug Programs

Table A1-1. Data sources and variables used in this analysis

| **Variable Name** | **Geography** | **Source (Variable ID)** |
| --- | --- | --- |
| Components of pharmacy desert definition | | |
| Population count (total and by age) | Census tract, block group | Decennial 2020 (P12_001N - P12_049N) |
| Median household income in 2021 dollars | Census tract, MSA, state | ACS 2021 (B19013_001E) |
| Number of individuals below the federal poverty line | Census tract | ACS 2021 (B17001_002E) |
| Number of individuals at or above the poverty line | Census tract | ACS 2021 (B17001_031E) |
| Number of individuals who do not own a vehicle | Census tract | ACS 2021 (B08201_001E) |
| Land area (used to calculated population density and urbanicity) | Census tract | Decennial 2020 (ALAND) |
| Characteristics of census tracts and pharmacies | | |
| Education: adults >25 with high school education or less | Census tract | ACS 2021 (B15001_001E) |
| Race/Ethnicity: Proportion of adults by self-described racial and ethnic identity | Census tract | ACS 2021 (B02001_001E – 009E) |
| Socioeconomic status: Proportion of individuals living below the FPL | Census tract | ACS 2021 (B17001_002E) |
| Elderly age: Proportion of adults age 65 or older | Census tract | ACS 2021 (B01001_001E- B01001_049E) |
| Health insurance (any): Proportion of adults with health insurance | Census tract | ACS 2021 (B27001) |
| Health insurance (public): Proportion of adults with public health insurance | Census tract | ACS 2021 (B27003) |
| English-speaking: Proportion of adults whose English-speaking ability is “not well” or “not at all” | Census tract | ACS 2021 (B16004_024E – 67E) |
| Physical disability status: Proportion of adults with an ambulatory difficulty | Census tract | ACS 2021 (B18105_006E – 032E)) |
| Inequality: Gini index by after-tax income | Census tract | ACS 2021 (B19083_001E) |
| Pharmacy ownership (independent, chain, franchise, government) | Point | NCPDP (Dispenser Class Code 01, 02, 05, 06) |
| Durable medical equipment offered | Point | NCPDP (DME Code 13, 14, 15, 16, 17, 18) |
| Walk-in clinic services offered | Point | NCPDP (Walk-in Clinic Code 19, 20, 21, 22) |
| Emergency services offered | Point | NCPDP (24h Emergency Service Code 23, 24, 25, 26, 27) |
| Immunizations offered | Point | NCPDP (Immunizations Provided Code 31, 32, 33) |
| Handicap accessibility offered | Point | NCPDP (Handicapped Accessible Indicator) |
| 340b relationships offered | Point | NCPDP (340B Status Code 36, 37, 38, 39) |

## 1.2 Geographic calculations

### 1.2.1 Coordinate reference systems

Pharmacy street addresses were geocoded (i.e., converted to latitude and longitude) using the R package ggmap and the Google Maps Application Programming Interface (API) which uses the World Geodetic System 1984 (WGS84) coordinate reference system (CRS) (EPSG: 4326).^1^ All polygons from the U.S. Census Bureau for state, county, MSA, tract, and block level data use the North American Datum of 1983 (NAD 83) CRS (EPSG: 4269). All geographic data were converted to the WGS84 CRS for mapping and analysis using the “st_crs” function in the R package “sf”. The mileage buffers around each pharmacy were calculated by converting to the Web Mercator projection (EPSG: 3857), which measures in meters. Distances of 804.672m, 1609.34m, 8046.720m, 16093.440m, respectively, were used to create the 0.5, 1, 5, and 10-mile radius buffers around pharmacies.

### 1.2.2 Urbanicity determination

Urbanicity was determined by dividing the total population count of a census tract by land area of the tract (both fields as provided in the 2020 Decennial Census). This population density was then used to create a categorical variable for urbanicity: density >5,000 people per square mile to indicate urban area, density between 1,000-5,000 people per square mile for suburban, and <1,000 people per square mile as indicative of rural area. Urbanicity was used to determine what the acceptable radius of a pharmacy is for the population in that tract.

An example visualization of the urban, suburban, and rural tracts along with varying pharmacy radii as examples can be seen in Figure A1-2. In the figure, light green denotes urban tracts, dark blue denotes suburban, and light blue denotes rural tracts as calculated based on population density. Black dots represent pharmacies and the orange circles around them represent the 0.5, 1, 5, or 10-mile radius around that pharmacy based on the urbanicity status of the tract the pharmacy is located in.

Figure A1-2. Example urbanicity of tracts and pharmacy radius example in Seattle.
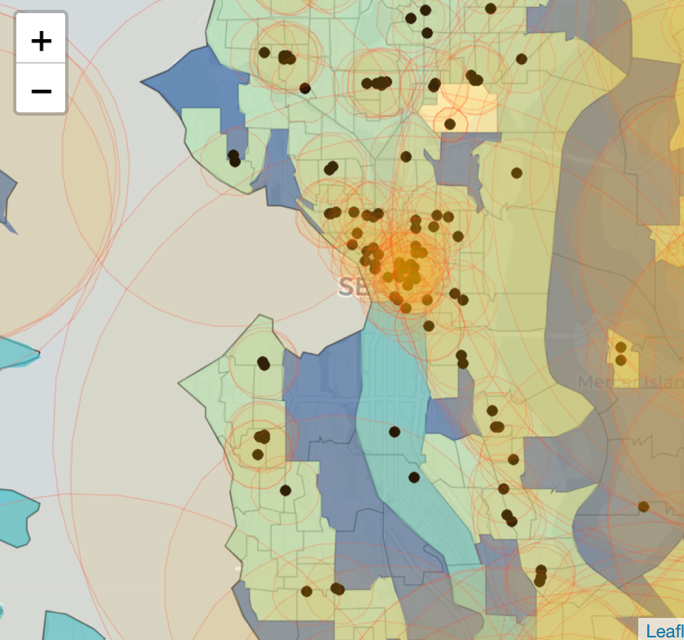


### 1.2.3 Population within pharmacy radius

To calculate the proportion of the population living within the acceptable radius of a pharmacy, we used areal interpolation at the block level. First, we identified the geographic centroid of every block in the U.S.. Then, we created a binary variable whether that block was within a 1-mile linear distance (as opposed to street travel distance) radius of any pharmacy using the “st_within” function of the “sf” R package. If the block centroid was inside, then the population of the block was counted as living within the pharmacy radius. We repeated this step for 0.5-mile, 5-, and 10-mile radius distances for all centroids and all pharmacies in the U.S. To calculate the proportion of a tract’s population living within the acceptable radius of a pharmacy for a given urbanicity of the tract, we summed the population of all the blocks that were inside the acceptable radius and divided that by the total population of the tract. For example, for a suburban census tract, we summed all the census blocks with centroids that were within the 5-mile radius of any pharmacy and divided it by the total population of that tract. In this way, the calculation allowed for access to pharmacies in other tracts, while preserving the acceptable access distance based on urbanicity of that specific tract.

This process is visualized in Figure A1-3, which highlights a hypothetical example of a census tract that is a pharmacy desert based on this method (outlined in red) and a non-pharmacy desert (outlined in black).

Figure A1-3. Visualization of the areal interpolation process


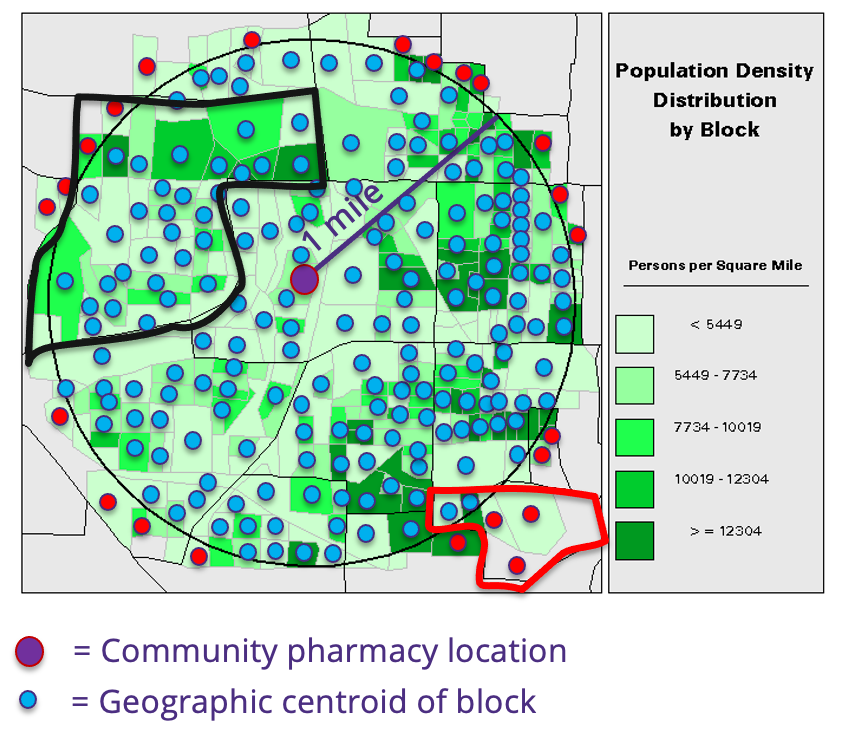


Based on original figure by Shepard E, et al. ESRI Conference Proceedings 1997. Available [here](https://proceedings.esri.com/library/userconf/proc97/proc97/to300/pap298/p298.htm).

## 1.3 Accessing source data and analysis code

The R code and a file of the processed data at the census tract level is available on the project GitHub page at this link <<https://github.com/rwitten1/Pharmacy-Deserts-Analysis>>. A public Tableau version of the pharmacy desert map is available here <<https://tinyurl.com/PharmacyDesertsMap2022>>. The NCPDP pharmacy address data is proprietary thus is not included in this public repository. The full R code used in dataset creation and analysis is available on this site as well. Researchers are welcome to contact the corresponding author with questions.

## 1.4 Additional study limitations

In addition to those listed in the main manuscript, we note several additional limitations and considerations for the reader in interpreting this analysis.

Design limitations: There is a need for a standardized technical definition of a “pharmacy desert” —nearly every study of pharmacy access uses a slightly different methodology, data, and definition.^2^ One key decision is the inclusion of an income-based component versus pure geographic access to pharmacies. As with the definitions of food deserts^3^ and HPSAs,^4,5^ the primary definition of *pharmacy desert* used in this analysis includes income in addition to spatial factors. We present results with and without the income-based component to enable comparison with other studies, though believe the inclusion of income and resource access on top of geography is important to consider and has theoretical foundations in Link and Phelan’s *fundamental cause* theory of health disparities.^6^ Determining a definition for the field is out of the scope of this analysis, though these results will be a useful input to the discussion.

Data limitations: Further, the characteristics of populations residing in pharmacy deserts are presented at the group level and thus preclude any inference about individual-level characteristics or risks. The NCPDP data is self-reported by pharmacies and may be less accurate than other objective measures of service availability. However, this was the most current data available to the research team, has been used in other studies,^7–9^ and aligns with findings from studies using alternative pharmacy data sources.

Analysis limitations: Our use of population density as a proxy for urban status presents several challenges. For example, tracts which contain schools or parks will have relatively lower average population density, and thus would be classified as suburban or rural even if they were surrounded by dense urban tracts. Other approaches to define urbanicity of a place include commuter patterns, inclusion in a designated metro or micropolitan area, urban/rural designation by the Census Bureau, and more.^10^ One alternative to using population density that was considered was using the US Department of Agriculture’s rural-urban commuter area (RUCA) codes, which are calculated at the census tract level and summarize a complex array of factors to create levels of urbanicity and which are used in a wide variety of analyses, including previous pharmacy desert analyses.^11,12^ However, the most recent RUCA codes at the time of this analysis were based on 2010 population levels, which is not current enough to be relevant for this analysis (2020 RUCA codes are expected to be available in Winter 2024). For this analysis, we chose population density because we needed a method that was available at the census tract level, able to indicate urban, suburban, and rural categories, and reflective of current population levels.^13^

Once urbanicity is defined, the corresponding acceptable threshold for distance (e.g., 1, 5, or 10 miles) and the definition of “appropriate” pharmacy-to-population relationships are inconsistently defined in the literature. Some studies have applied a binary 1-mile (urban) vs 10-mile (rural) access radius classification,^14^ others use 10 miles regardless of urbanicity,^15^ others use 2-, 5-, or 10-miles^9^ as based on the CMS requirements for minimum pharmacy insurance benefits^16^, while still others use pharmacy-to-population ratios instead. We chose 1-, 5-, and 10-mile radii to merge the most common pharmacy desert definition (1- and 10-mile) with the CMS pharmacy requirement of 2, 5, and 10-mile because we wanted results to be based in policy though agree with other analyses that 2 miles is not a reasonable radius in urban areas.

Lastly, due to computing resource constraints, in this analysis we do not calculate road travel distance or time in transit in evaluating distance to pharmacies. Instead, we used linear distance, which likely underestimates the true distance and effort required to access a pharmacy.^17^

Statistical limitations: In this analysis we are examining bivariate associations at the population level, which has important limitations. One, without any individual data on inhabitants in each tract we can only make inferences about tract-level average characteristics, not characteristics of individuals who live in pharmacy deserts. Two, these are associations rather than causal links, and do not reveal any information about why these patterns exist. Three, while many of the average characteristic differences are *statistically* significant between pharmacy deserts and non-pharmacy deserts, the magnitude of those differences may not be “significant” in their scientific implications. For example, the proportion of individuals with a high school education or less is 33% in pharmacy deserts versus 28% in non-pharmacy deserts. While statistically distinct, even when adjusting our tests for multiple comparisons, there may not be a practical difference in, say, designing policy solutions based on the fact that 5% more of the population has lower educational background in pharmacy deserts compared to non-deserts.

# Supplement A2: Results supplement

## 2.1 Population living in pharmacy deserts by state

Figure A2-1. Proportion of adult population living in pharmacy deserts in each state


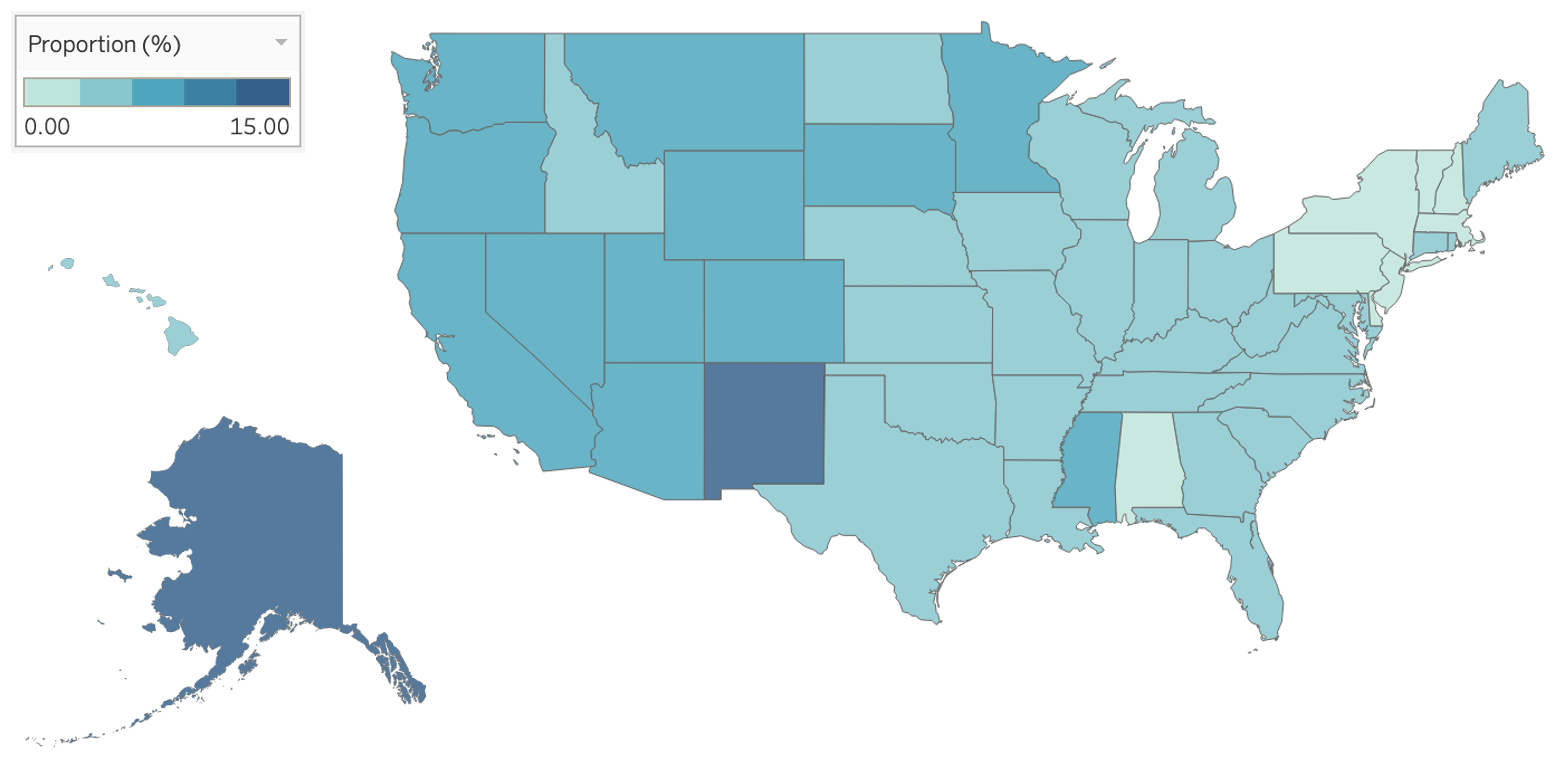


Table A2-1. Population living in pharmacy deserts by state

| State | Proportion of the Adult Population Living in Pharmacy Desert (%) | Proportion of the Total Population Living in Pharmacy Desert (%) | Total Number of People Living in a Pharmacy Desert (N) |
| --- | --- | --- | --- |
| New Mexico | 14.89 | 15.18 | 320,932 |
| Alaska | 14.77 | 15.47 | 113,488 |
| Arizona | 8.64 | 8.99 | 640,797 |
| Colorado | 8.60 | 8.60 | 495,596 |
| South Dakota | 8.28 | 8.90 | 78,899 |
| Montana | 8.17 | 8.38 | 90,817 |
| Oregon | 7.92 | 8.04 | 340,621 |
| Washington | 7.62 | 7.72 | 594,498 |
| Mississippi | 7.56 | 7.40 | 219,252 |
| Minnesota | 7.09 | 7.17 | 409,101 |
| Utah | 6.82 | 6.44 | 210,351 |
| Nevada | 6.33 | 6.59 | 204,434 |
| Wyoming | 6.22 | 5.95 | 34,348 |
| California | 6.17 | 6.43 | 2,535,219 |
| Texas | 5.74 | 5.79 | 1,683,909 |
| North Dakota | 5.47 | 5.40 | 42,070 |
| Oklahoma | 5.37 | 5.35 | 211,562 |
| Maine | 5.26 | 5.21 | 70,955 |
| Connecticut | 5.24 | 5.36 | 192,996 |
| Louisiana | 5.16 | 5.07 | 235,950 |
| Missouri | 5.07 | 5.08 | 312,678 |
| Ohio | 4.96 | 5.01 | 590,536 |
| Virginia | 4.89 | 4.85 | 417,496 |
| Idaho | 4.87 | 4.62 | 85,030 |
| Maryland | 4.86 | 5.01 | 308,608 |
| West Virginia | 4.81 | 4.69 | 84,134 |
| Kentucky | 4.63 | 4.59 | 206,389 |
| Kansas | 4.47 | 4.40 | 129,344 |
| Wisconsin | 4.47 | 4.52 | 266,395 |
| Illinois | 4.43 | 4.46 | 571,740 |
| Indiana | 4.20 | 4.23 | 286,337 |
| Michigan | 4.14 | 4.22 | 423,339 |
| Nebraska | 4.03 | 4.19 | 82,053 |
| Hawaii | 3.87 | 4.11 | 59,847 |
| Arkansas | 3.84 | 3.70 | 111,396 |
| Iowa | 3.44 | 3.35 | 106,671 |
| Florida | 3.42 | 3.48 | 749,103 |
| Georgia | 3.41 | 3.47 | 371,241 |
| South Carolina | 3.38 | 3.26 | 166,586 |
| Tennessee | 3.33 | 3.42 | 235,886 |
| Rhode Island | 3.25 | 3.32 | 36,479 |
| North Carolina | 3.02 | 2.94 | 306,247 |
| Massachusetts | 2.93 | 2.93 | 205,691 |
| Delaware | 2.60 | 2.61 | 25,868 |
| Alabama | 2.52 | 2.40 | 120,601 |
| Pennsylvania | 2.51 | 2.52 | 327,033 |
| District of Columbia | 1.98 | 1.91 | 13,179 |
| Vermont | 1.80 | 1.77 | 11,406 |
| New York | 1.74 | 1.83 | 369,336 |
| New Jersey | 1.05 | 1.15 | 106,706 |
| New Hampshire | 0.86 | 0.86 | 11,778 |

## 2.2 Pharmacy accessibility results

Figure A2-2. Pharmacy desert locations in the United States


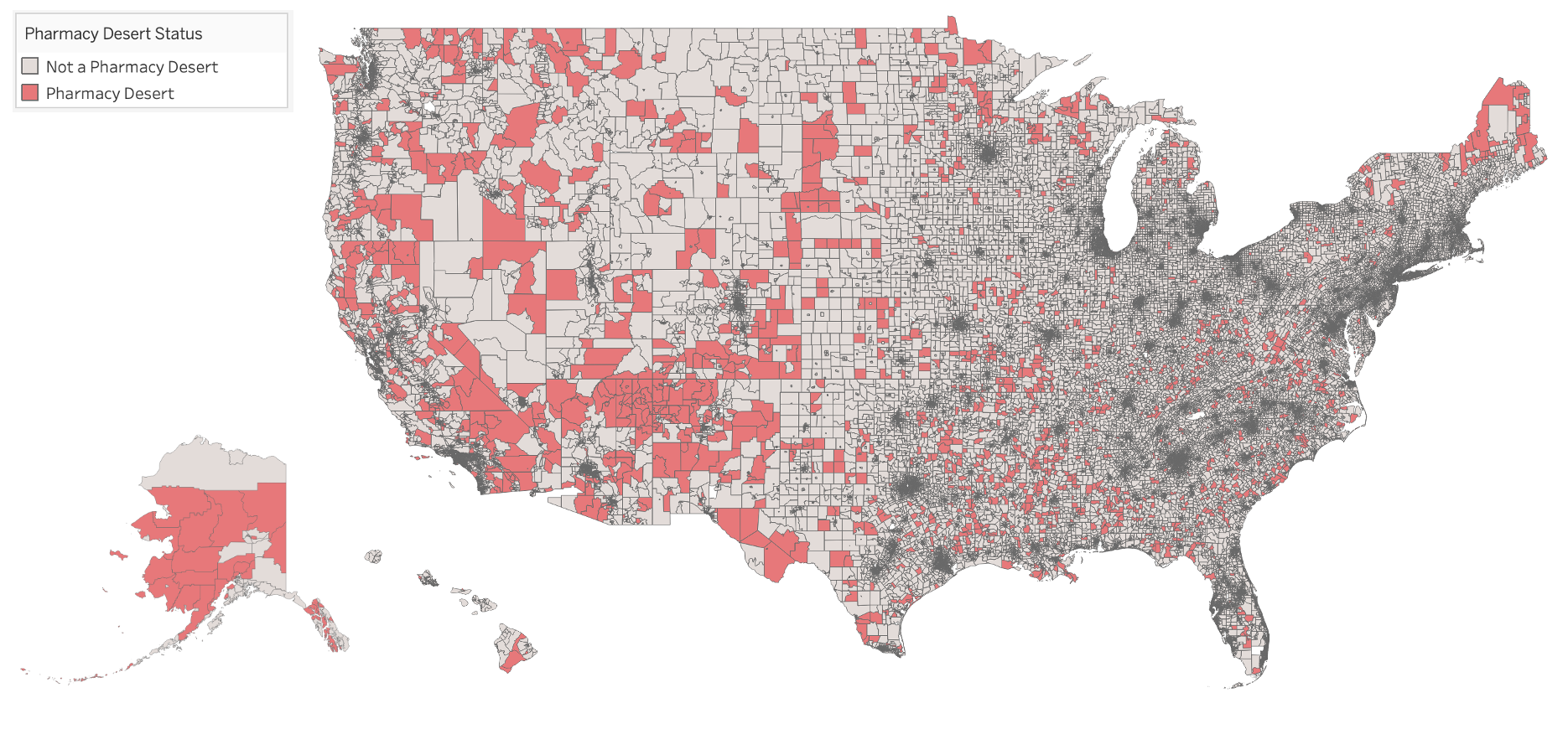


Figure A2-3. Pharmacy desert locations in select urban cities throughout the US


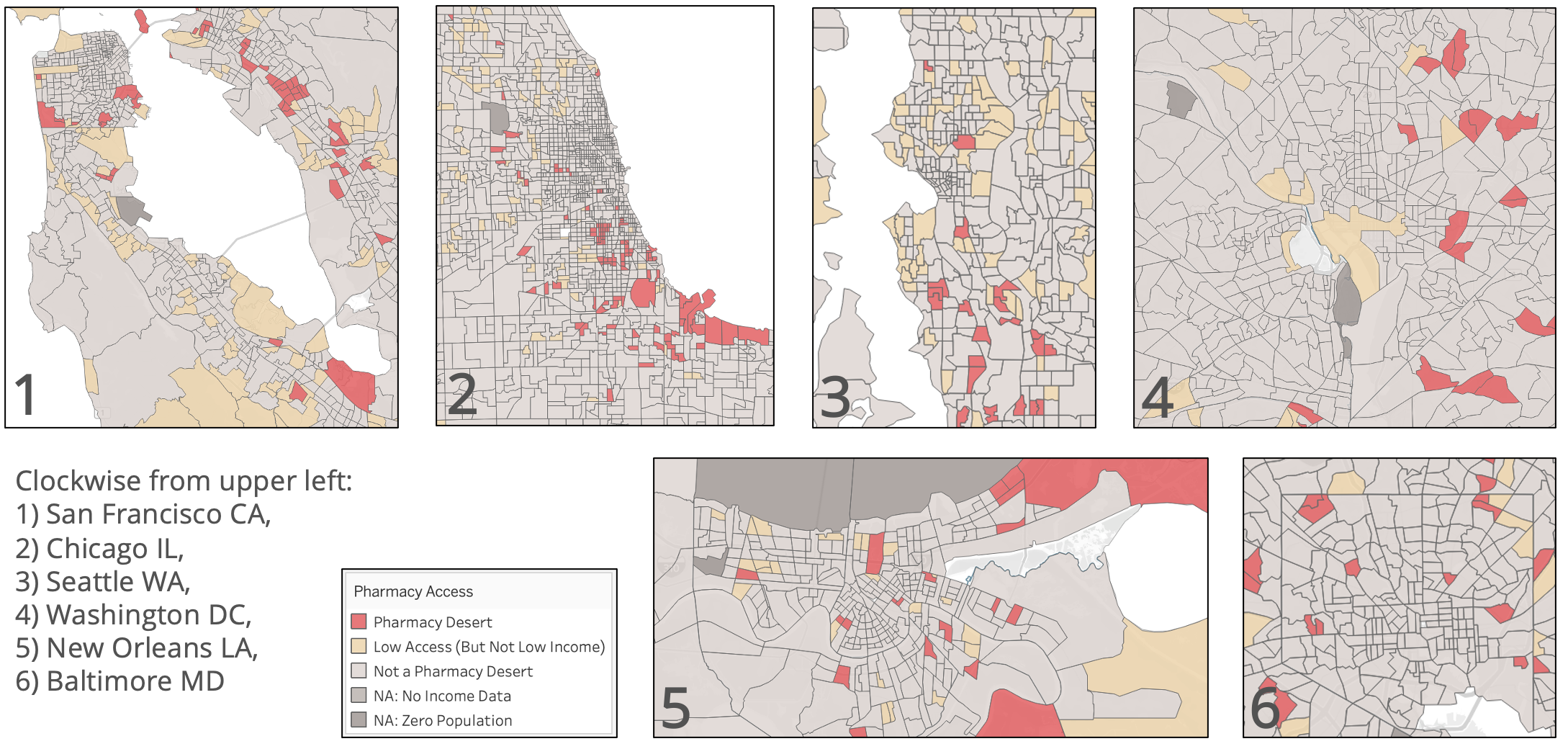


Figure A2-4. Pharmacy locations in the US.


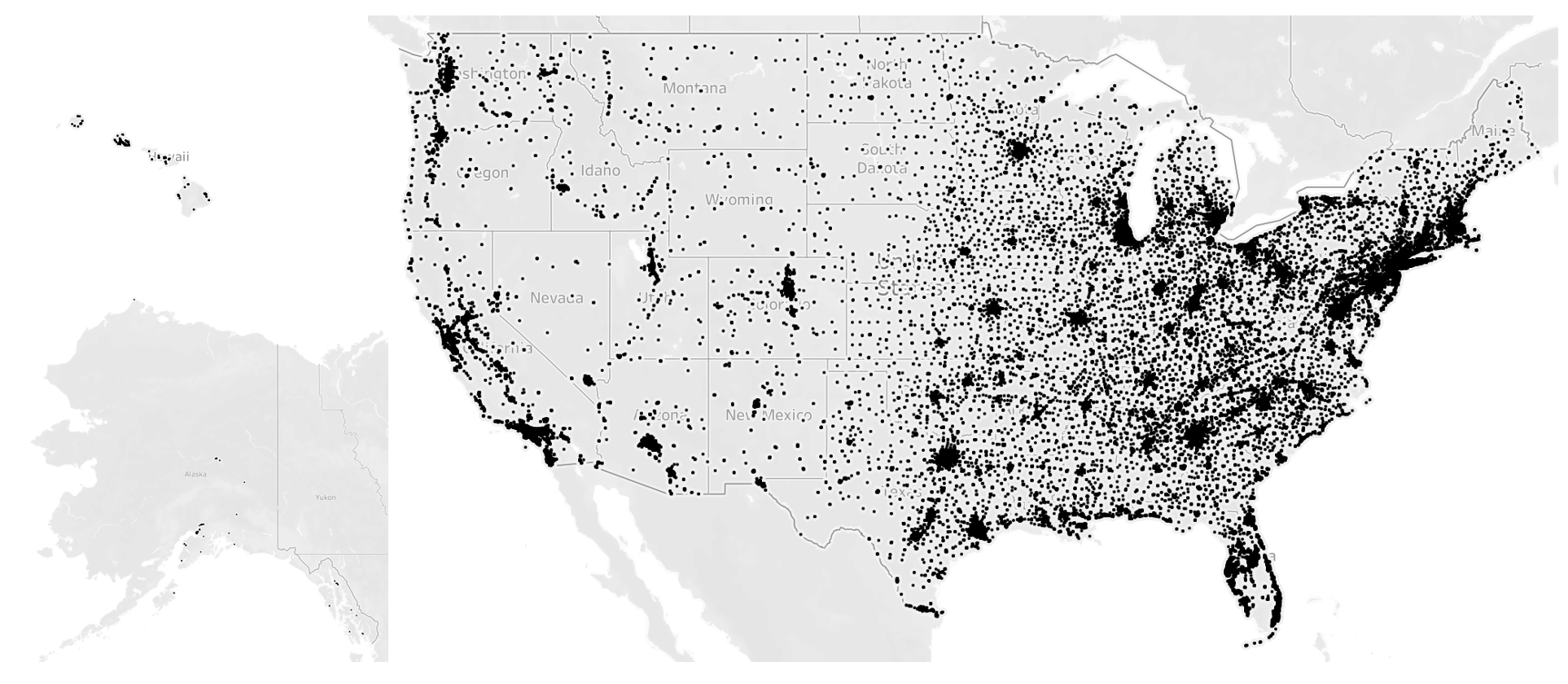


Figure A2-5. Pharmacy counts by census tract


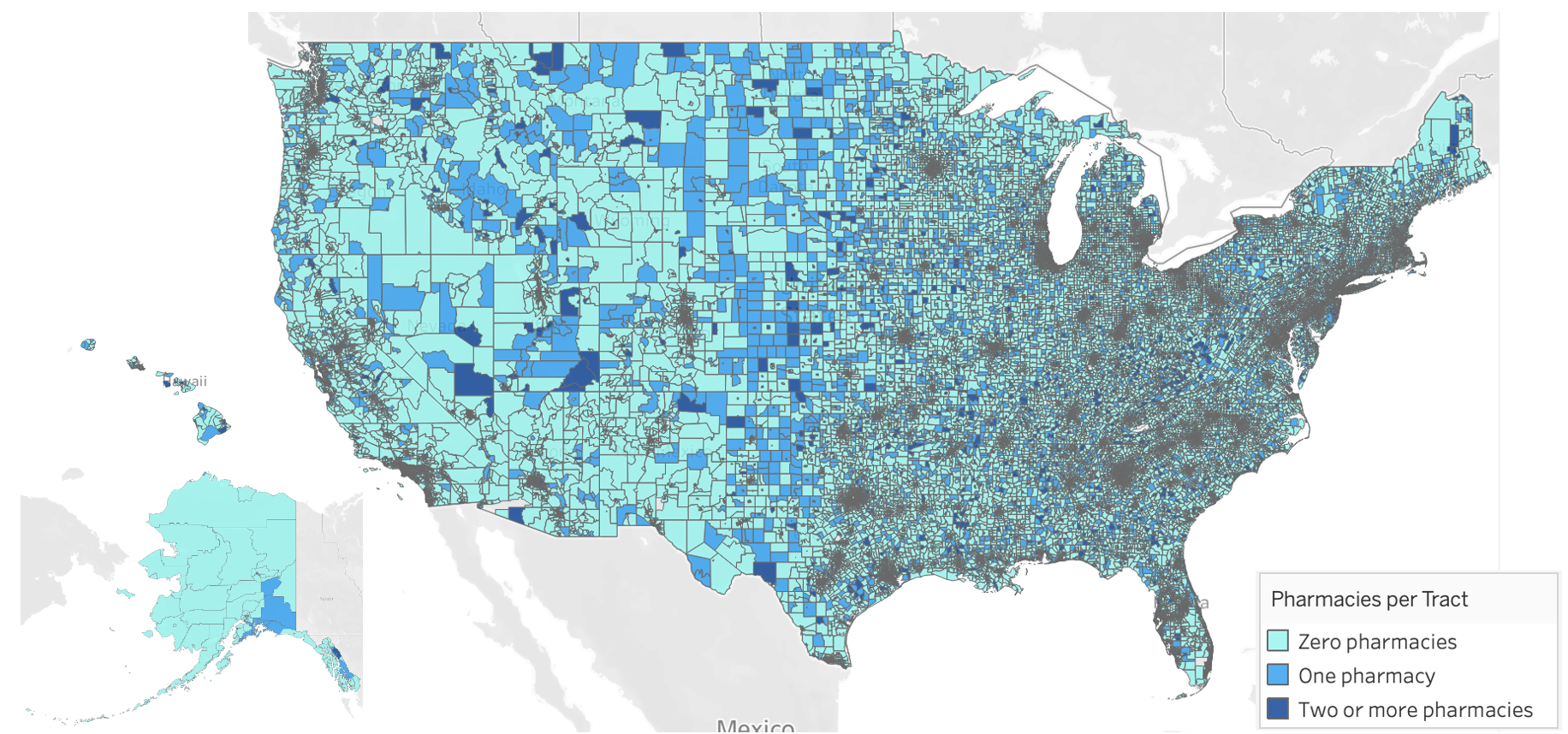


## 2.3 Characteristics of populations and pharmacies in pharmacy deserts

Table A2-2. Pharmacy desert characteristics stratified by urbanicity.

|  | **Urban** | | **Suburban** | | **Rural** | |
| --- | --- | --- | --- | --- | --- | --- |
| **Mean (SD)** | **Pharmacy Desert** (N=2,692) | **Not Pharmacy Desert** (N=19,972) | **Pharmacy Desert** (N=204) | **Not Pharmacy Desert** (N=30,264) | **Pharmacy Desert** (N=1,783) | **Not Pharmacy Desert** (N=28,487) |
| **Prop. Below FPL** | 25.3% (13.9) | 15.4% (12.1) | 31.9% (22.2) | 12.7% (11.4) | 22.7% (13.4) | 11.7% (8.63) |
| **Median Income** | $47,100 ($16,100) | $76,800 ($37,700) | $44,700 ($21,500) | $79,100 ($40,700) | $45,500 ($11,700) | $72,000 ($31,500) |
| **Prop. High School Education or Less** | 30.5% (9.69) | 24.0% (10.9) | 32.3% (12.0) | 25.6% (0.11.0) | 37.4% (0.09.78) | 32.3% (10.6) |
| **Prop. With No Health Insurance** | 16.3% (11.9) | 11.0% (09.62) | 12.0% (12.6) | 9.69% (8.69) | 13.9% (9.79) | 9.33% (7.48) |
| **Prop. With Public Health Insurance** | 38.2% (15.6) | 33.7% (14.0) | 42.2% (22.4) | 34.1% (13.0) | 47.5% (13.3) | 38.0% (11.4) |
| **Pharmacies per Tract (N (%))** |  |  |  |  |  |  |
| Zero pharmacies | 2,537 (94.2%) | 10,266 (51.4%) | 198 (97.1%) | 15,065 (49.8%) | 1,686 (94.6%) | 17,918 (62.9%) |
| One pharmacy | 132 (4.9%) | 5,912 (29.6%) | 5 (2.5%) | 8,238 (27.2%) | 87 (4.9%) | 6,472 (22.7%) |
| Two or more pharmacies | 23 (0.9%) | 3,794 (19.0%) | 1 (0.5%) | 6,961 (23.0%) | 10 (0.6%) | 4,097 (14.4%) |
| **Prop. Do Not Speak English** | 7.91% (10.4) | 6.64% (9.99) | 4.39% (10.2) | 2.40% (5.30) | 2.58% (6.87) | 1.30% (3.85) |
| **Prop. Ambulatory Disability** | 9.07% (5.57) | 7.21% (4.66) | 10.4% (8.21) | 8.05% (4.87) | 12.5% (6.70) | 9.45% (5.06) |
| **Prop. Older Adult (65+)** | 11.3% (7.84) | 13.4% (7.11) | 15.3% (13.3) | 16.9% (9.42) | 20.7% (10.0) | 19.5% (8.29) |
| **Prop. NH, White** | 32.8% (26.4) | 39.2% (27.6) | 54.4% (29.2) | 60.2% (26.8) | 63.8% (31.2) | 77.5% (21.7) |
| **Prop. NH, Black** | 25.3% (28.6) | 16.0% (22.7) | 17.0% (22.7) | 14.9% (22.1) | 9.88% (18.9) | 7.79% (15.1) |
| **Prop. NH, Asian** | 4.88% (8.03) | 10.5% (14.3) | 2.67% (5.55) | 5.11% (8.20) | 0.962% (4.40) | 1.72% (4.43) |
| **Prop. NH, AIAN** | 0.45% (1.61) | 0.26% (0.86) | 1.33% (7.52) | 0.362% (1.46) | 8.33% (23.1) | 0.84% (4.29) |
| **Prop. NH, 2 or More Races** | 3.24% (3.33) | 3.42% (3.38) | 2.93% (2.95) | 3.37% (3.11) | 2.69% (3.30) | 2.75% (3.00) |
| **Prop. NH, Other Race** | 0.41% (1.13) | 0.54% (1.49) | 0.20% (0.71) | 0.36% (1.03) | 0.23% (0.85) | 0.23% (0.78) |
| **Prop. Hispanic, White Race** | 15.3% (16.9) | 12.7% (13.7) | 10.9% (15.5) | 7.94% (11.3) | 7.23% (13.5) | 4.70% (8.82) |
| **Prop. Hispanic, Black Race** | 0.56% (1.24) | 0.71% (1.69) | 0.52% (2.00) | 0.31% (0.96) | 0.12% (0.59) | 0.13% (0.73) |
| **Prop. Hispanic, AIAN Race** | 0.46% (1.27) | 0.37% (1.04) | 0.25% (0.76) | 0.20% (0.76) | 0.38% (1.35) | 0.13% (0.61) |
| **Prop. Hispanic, 2 or More Races** | 6.09% (7.28) | 5.77% (6.63) | 3.86% (6.66) | 3.36% (5.06) | 2.80% (5.61) | 1.97% (3.97) |
| **Prop. Hispanic, Other Race** | 10.0% (0.12.5) | 10.1% (13.0) | 5.71% (11.3) | 3.64% (6.34) | 3.40% (8.20) | 2.06% (4.95) |

**Characteristics of populations living in low-access tracts**

Table A2-3 summarizes characteristics of populations of tracts that were designated low-access but did not meet the low-income criterion of the pharmacy desert definition.

Table A2-3. Characteristics of populations in low-access tracts

|  | **Low Access (N=12,646)** | **Not Low Access (N=71,202)** | ***p*-value** |
| --- | --- | --- | --- |
| **Urbanicity and access (n (%))** |  |  |  |
| **Urbanicity of census tract** |  |  |  |
| Urban | 5,790 (45.8%) | 16,947 (23.8%) | <0.001 |
| Suburban | 652 (5.2%) | 29,887 (42.0%) |  |
| Rural | 5,959 (47.1%) | 24,360 (34.2%) |  |
| NA: No Population | 245 (1.9%) | 8 (0.0%) |  |
| **Pharmacies per census tract** |  |  |  |
| Zero pharmacies in the tract | 721 (5.7%) | 20,149 (28.3%) | <0.001 |
| One pharmacy in the tract | 116 (0.9%) | 14,786 (20.8%) |  |
| Two or more pharmacies | 11,809 (93.4%) | 36,267 (50.9%) |  |
| **Social characteristics (mean (SD))** |  |  |  |
| Prop. Below FPL | 14.7 (0.12.4) | 13.5 (0.11.1) | <0.001 |
| Median Household Income | $69,100 ($32,000) | $75,200 ($37,400) | <0.001 |
| Prop. With HS Educ. Or Less | 30.6 (0.11.5) | 27.5 (0.11.4) | <0.001 |
| Prop. With No Health Insurance | 11.0 (0.09.40) | 10.0 (0.08.70) | <0.001 |
| Prop. With Public Health Insurance | 37.0 (0.14.2) | 35.5 (0.12.9) | <0.001 |
| Prop. Do Not Speak English | 3.48 (0.07.26) | 3.18 (0.06.95) | <0.001 |
| **Demographic characteristics (mean (SD))** |  |  |  |
| Prop. With Ambulatory Disability | 8.62 (5.57) | 8.43 (5.00) | <0.001 |
| Prop. Older Adult (Age 65+) | 16.3 (9.49) | 16.9 (8.80) | <0.001 |
| **Race and ethnicity** |  |  |  |
| Prop. NH, White | 59.7 (31.3) | 60.3 (29.4) | 0.045 |
| Prop. NH, Black | 11.2 (19.8) | 13.3 (21.0) | <0.001 |
| Prop. NH, Asian | 4.42 (9.44) | 5.27 (9.71) | <0.001 |
| Prop. NH, AIAN | 1.88 (9.82) | 0.470 (2.58) | <0.001 |
| Prop. NH, 2 or More Races | 3.15 (3.41) | 3.15 (3.12) | 0.978 |
| Prop. NH, Other Race | 0.299 (0.88) | 0.369 (1.12) | <0.001 |
| Prop. Hispanic, White Race | 9.19 (13.3) | 8.03 (11.7) | <0.001 |
| Prop. Hispanic, Black Race | 0.281 (1.15) | 0.362 (1.15) | <0.001 |
| Prop. Hispanic, AIAN Race | 0.288 (0.954) | 0.220 (0.82) | <0.001 |
| Prop. Hispanic, 2 or More Races | 3.93 (5.99) | 3.47 (5.37) | <0.001 |
| Prop. Hispanic, Other Race | 5.31 (9.54) | 4.77 (8.89) | <0.001 |

**Pharmacy services details**

Table A2-4 summarizes characteristics of pharmacies in pharmacy desert communities versus not in pharmacy desert communities with more granular service categories than Table 2 in the main manuscript.

Table A2-4. Characteristics of pharmacies located in pharmacy deserts

|  | **Pharmacy Desert (N=294)** | **Not Pharmacy Desert (N=60175)** | ***p*-value*** |
| --- | --- | --- | --- |
| **Urbanicity** |  |  |  |
| Urban | 179 (60.9%) | 15,986 (26.6%) | <0.001 |
| Suburban | 8 (2.7%) | 26,922 (44.7%) |  |
| Rural | 107 (36.4%) | 17,206 (28.6%) |  |
| NA: No Population | 0 (0%) | 61 (0.1%) |  |
| **Pharmacy Ownership** |  |  |  |
| Independent | 121 (41.2%) | 22,010 (36.6%) | <0.001 |
| Chain | 165 (56.1%) | 37,371 (62.1%) |  |
| Franchise | 3 (1.0%) | 659 (1.1%) |  |
| Government | 5 (1.7%) | 135 (0.2%) |  |
| **Immunization Services Availability** |  |  |  |
| No on-site immunizations | 73 (24.8%) | 11,665 (19.4%) | <0.001 |
| Immunization services at select dates and times | 34 (11.6%) | 3,266 (5.4%) |  |
| Immunization services during business hours | 187 (63.6%) | 45,244 (75.2%) |  |
| **ADA Accessibility** |  |  |  |
| Not ADA accessible | 4 (1.4%) | 746 (1.2%) | 1.000 |
| **340b Status Category** |  |  |  |
| No 340B relationships | 219 (74.5%) | 50,943 (84.7%) | <0.001 |
| Not owned by 340B entity but contracts to covered entities | 53 (18.0%) | 7,417 (12.3%) |  |
| Owned by 340B entity but also serves non-eligible patients | 21 (7.1%) | 1,692 (2.8%) |  |
| Owned by 340B entity and only serves eligible patients | 1 (0.3%) | 123 (0.2%) |  |
| **Multidose Packaging Availability** |  |  |  |
| No multidose compliance packaging | 221 (75.2%) | 48,119 (80.0%) | <0.001 |
| Multidose compliance packaging to assisted living facilities only | 31 (10.5%) | 2,841 (4.7%) |  |
| Multidose compliance packaging to all | 42 (14.3%) | 9,215 (15.3%) |  |
| **Emergency Services 24 Hours Availability** |  |  |  |
| No 24h emergency service | 212 (72.1%) | 42,334 (70.4%) | <0.001 |
| 24h emergency remote pharmacist (call center) | 38 (12.9%) | 10,706 (17.8%) |  |
| 24h emergency remote pharmacist (local) | 24 (8.2%) | 5,431 (9.0%) |  |
| 24h emergency pharmacist with in-person access | 19 (6.5%) | 1551 (2.6%) |  |
| 24h in-person emergency pharmacist service | 1 (0.3%) | 153 (0.3%) |  |
| **Walk-in Clinic Available** |  |  |  |
| No walk-in clinic | 254 (86.4%) | 56,252 (93.5%) | <0.001 |
| Walk-in clinic with limited services, mid-level professional | 21 (7.1%) | 2,053 (3.4%) |  |
| Walk-in clinic with limited services, licensed physician | 12 (4.1%) | 1,489 (2.5%) |  |
| Onsite emergency room | 7 (2.4%) | 381 (0.6%) |  |
| **Compounding Pharmacy Category** |  |  |  |
| No compounding services | 122 (41.5%) | 22,945 (38.1%) | 0.070 |
| Basic non-sterile compounding | 160 (54.4%) | 35,591 (59.1%) |  |
| Complex non-sterile compounding | 7 (2.4%) | 1,263 (2.1%) |  |
| Low complexity sterile compounding | 4 (1.4%) | 241 (0.4%) |  |
| High complexity sterile compounding | 1 (0.3%) | 135 (0.2%) |  |
| **DME Availability** |  |  |  |
| No DME offered | 75 (25.5%) | 13,433 (22.3%) | 0.034 |
| DME off-the-shelf, non-custom, unaccredited | 43 (14.6%) | 7,609 (12.6%) |  |
| DME full range and custom, unaccredited | 4 (1.4%) | 1,656 (2.8%) |  |
| DME for pharmaceuticals and diabetic testing, accredited | 98 (33.3%) | 17,283 (28.7%) |  |
| DME off-the-shelf, non-custom, accredited | 37 (12.6%) | 9,785 (16.3%) |  |
| DME full range and custom, accredited | 37 (12.6%) | 10409 (17.3%) |  |

Footnotes: * *p-*value is from a t-test for continuous variables and a chi-squared test for categorical variables, all adjusted for multiple comparisons using the Benjamini-Hochberg correction.

# Supplemental Materials References

1. Kahle D, Wickham H. ggmap: Spatial Visualization with ggplot2. Published online 2013:144-161.

2. Fernandes BD, Foppa AA, Almeida PHRF, Lakhani A, Lima T de M. Application and utility of geographic information systems in pharmacy specific health research: A scoping review. *Research in Social and Administrative Pharmacy*. 2022;18(8):3263-3271. doi:10.1016/j.sapharm.2021.11.004

3. Liese AD, Hibbert JD, Ma X, Bell BA, Battersby SE. Where Are the Food Deserts? An Evaluation of Policy-Relevant Measures of Community Food Access in South Carolina. *J Hunger Environ Nutr*. 2014;9(1):16-32. doi:10.1080/19320248.2013.873009

4. Wang F, Luo W. Assessing spatial and nonspatial factors for healthcare access: towards an integrated approach to defining health professional shortage areas. *Health Place*. 2005;11(2):131-146. doi:10.1016/J.HEALTHPLACE.2004.02.003

5. Murphy EM, West L, Jindal N. Pharmacist provider status: Geoprocessing analysis of pharmacy locations, medically underserved areas, populations, and health professional shortage areas. *Journal of the American Pharmacists Association*. 2021;61(6):651-660.e1. doi:10.1016/J.JAPH.2021.08.021

6. Link BG, Phelan JO. Social Conditions as Fundamental Causes of Disease. *J Health Soc Behav*. 1995;Spec:80-94.

7. Guadamuz JS, Alexander GC, Zenk SN, Qato DM. Assessment of Pharmacy Closures in the United States from 2009 Through 2015. *JAMA Intern Med*. 2020;180(1):157-160. doi:10.1001/jamainternmed.2019.4588

8. Hernandez I, Tang S, Morales J, et al. Role of Independent vs. Chain Pharmacies in Providing Pharmacy Access: A Nationwide Individual-Level Geographic Information Systems Analysis. *Health Affairs Scholar*. Published online June 20, 2023. doi:10.1093/HASCHL/QXAD003

9. Berenbrok LA, Tang S, Gabriel N, et al. Access to Community Pharmacies: A Nation-Wide Geographic Information Systems Cross-sectional Analysis. *Journal of the American Pharmacists Association*. 2022;0(0). doi:10.1016/J.JAPH.2022.07.003

10. Hall SA, Kaufman JS, Ricketts TC. Defining urban and rural areas in U.S. epidemiologic studies. *Journal of Urban Health*. 2006;83(2):162-175. doi:10.1007/S11524-005-9016-3/METRICS

11. Pourebrahim N, Shah P, VoPham T, et al. Time and geographic variations in human papillomavirus vaccine uptake in Washington state. *Prev Med (Baltim)*. 2021;153:106753. doi:10.1016/J.YPMED.2021.106753

12. Wittenauer R, Shah P, Bacci JL, Stergachis A. Pharmacy deserts and COVID-19 risk at the census tract level in the State of Washington. *Vaccine X*. Published online October 2022:100227. doi:10.1016/J.JVACX.2022.100227

13. Sharareh N, Zheutlin AR, Qato DM, Guadamuz J, Bress A, Vos RO. Access to community pharmacies based on drive time and by rurality across the contiguous United States. *Journal of the American Pharmacists Association*. 2024;0(0). doi:10.1016/j.japh.2024.01.004

14. Qato DM, Daviglus ML, Wilder J, Lee T, Qato D, Lambert B. ‘Pharmacy Deserts’ Are Prevalent In Chicago’s Predominantly Minority Communities, Raising Medication Access Concerns. *Health Aff*. 2014;33(11):1958-1965. doi:10.1377/hlthaff.2013.1397

15. Urick BY, Adams JK, Bruce MR. State Telepharmacy Policies and Pharmacy Deserts. *JAMA Netw Open*. 2023;6(8):e2328810. doi:10.1001/jamanetworkopen.2023.28810

16. CMS. *Prescription Drug Benefit Manual 5: Benefits and Beneficiary Protections*.; 2011. Accessed November 2, 2023. https://www.cms.gov/medicare/prescription-drug-coverage/prescriptiondrugcovcontra/downloads/memopdbmanualchapter5_093011.pdf

17. Ying X, Kahn P, Mathis WS. Pharmacy deserts: More than where pharmacies are. *J Am Pharm Assoc (2003)*. 2022;62(6):1875-1879. doi:10.1016/J.JAPH.2022.06.016
